# Phytotoxic Activity of Metabolites Isolated from *Rutstroemia sp.n.*, the Causal Agent of Bleach Blonde Syndrome on Cheatgrass (*Bromus tectorum*)

**DOI:** 10.3390/molecules23071734

**Published:** 2018-07-16

**Authors:** Marco Masi, Susan Meyer, Marcin Górecki, Gennaro Pescitelli, Suzette Clement, Alessio Cimmino, Antonio Evidente

**Affiliations:** 1 Dipartimento di Scienze Chimiche, Università di Napoli “Federico II”, Complesso Universitario Monte S. Angelo, Via Cintia 4, 80126 Napoli, Italy; marco.masi@unina.it (M.M.); alessio.cimmino@unina.it (A.C.); 2 US Forest Service Rocky Mountain Research Station, Shrub Sciences Laboratory, 735 North 500 East, Provo, UT 84606, USA; semeyer@xmission.com (S.M.); sclement@fs.fed.us (S.C.); 3 Dipartimento di Chimica e Chimica Industriale, Università di Pisa, Via Moruzzi 13, 56124 Pisa, Italy; marcin.gorecki@icho.edu.pl (M.G.); gennaro.pescitelli@unipi.it (G.P.); 4 Institute of Organic Chemistry, Polish Academy of Sciences, ul. Kasprzaka 44/52, 01-224 Warsaw, Poland

**Keywords:** *Bromus tectorum*, *Rutstroemia sp.n.*, phytotoxic metabolites, immersion bioassay, 9-*O*-methylfusarubin and terpestacin

## Abstract

A fungal pathogen soon to be described as *Rutstroemia capillus-albis* (Rutstroemiaceae, Helotiales, Leotiomycetes) has been identified as the causal agent of ‘bleach blonde syndrome’ on the invasive annual grass weed *Bromus tectorum* (cheatgrass) in western North America. This apparently common but previously undescribed disease causes premature senescence and sterility, but does not affect seed germination or seedling emergence and growth. This study investigated whether the new species produces phytotoxins that could be implicated in pathogenesis. The compounds 9-*O*-methylfusarubin, 9-*O*-methylbostrycoidin, 5-*O*-methylnectriafurone, *trans*-methyl-*p*-coumarate and terpestacin were isolated from the solid culture of this fungus. The undescribed absolute stereochemistry at C-3 of 9-*O*-methylfusarubin and at C-1’ of 5-*O*-methylnectriafurone were assigned by applying electronic and vibrational circular dichroism (ECD and VCD) combined with computational methods and the advanced Mosher’s method, respectively. The first three listed compounds are naphtoquinone pigments, while terpestacin is a sesterterpene, and *trans*-methyl-*p*-coumarate could be the product of an unusual fungal phenylpropanoid biosynthesis pathway. In a juvenile plant immersion bioassay, both 9-*O*-methylfusarubin and terpestacin proved to be highly toxic at 10^−4^ M, causing wilting and plant death within 10 days. This finding suggests that these two compounds could play a role in pathogenesis on *B. tectorum*.

## 1. Introduction

The invasive winter annual grass weed *Bromus tectorum* (cheatgrass) is dramatically altering the semi-arid shrubland ecosystems in the western USA. Increasing wildfire frequency and intensity has resulted in near-monocultures of this weed over very large areas [1,2]. A frequent occurrence in some areas heavily invaded by *B. tectorum* is periodic ‘cheatgrass die-off’ or complete stand failure. This poorly understood phenomenon is apparently caused by a complex interaction among multiple soilborne fungal pathogens [3]. To help elucidate the ‘die-off’ phenomenon, the ability of some of these pathogens to produce phytotoxins was investigated. From the solid cultures of *Pyrenophora semeniperda* (Brittlebank and Adams) Shoemaker [4,5], a large quantity of cytotoxic cytochalasin B was isolated, along with smaller quantities of related cytochalasans [6]. Successively, the known abscisic acid was also isolated together with three new phytotoxic sesquiterpenoids (named pyrenophoric acid and pyrenophoric acids B and C) from the same cultures [7,8]. In a cheatgrass seedling bioassay, all these compounds were able to induce growth suppression of both radicles and coleoptiles [6,7,8]. From the potato dextrose broth culture of *P. semeniperda*, a new γ-lactam, named spirostaphylotrichin W, was isolated together with some known spirostaphylotrichins. Some of these compounds caused necrosis in leaf puncture bioassays while having little effect on cheatgrass seedlings [9,10].

Recently, acuminatopyrone, blumenol A, chlamydosporol, isochlamydosporol, ergosterol and 4-hydroxybenzaldehyde were isolated from a *Fusarium* strain belonging to the *Fusarium tricinctum* species complex grown on cheatgrass seed culture. In a *B. tectorum* seedling bioassay, 4-hydroxybenzaldehyde proved to be the most active compound [11].

During investigation of the die-off phenomenon, epidemic levels of a previously undescribed disease on *B. tectorum* were noted in some years. This disease, named the ’bleach blonde syndrome’, causes premature senescence, sterility, and death at the heading stage. It is not a direct cause of stand failure, but may be a factor that predisposes a *B. tectorum* stand to emergence failure in a subsequent year [3]. Pathogenicity tests combined with molecular genetic analysis demonstrated that the causal agent of this disease is a new fungus of the genus *Rutstroemia* related to the dollar spot pathogen of turf (*‘Sclerotinia’ homoeocarpa*). This fungus has been described as *Rutstroemia capillus-albis* (Rutstroemiaceae, Helotiales, Leotiomycetes) [12]. 

As secondary metabolite production in the genus *Rutstroemia* has never been examined and is poorly studied in the Leotiomycetes in general, a study was undertaken to determine whether secondary metabolites that could play a role in pathogenesis were produced by this fungus. Furthermore, their potential application as natural herbicides was also evaluated for the biological control of cheatgrass.

This paper reports the isolation of five secondary metabolites produced by the newly described plant pathogen *R. capillus-albis*, as well as their phytotoxic activity against *B. tectorum*.

## 2. Material and Methods

### 2.1. General Experimental Procedures

Optical rotations were measured in MeOH on a Jasco P-1010 digital polarimeter (Jasco, Tokyo, Japan). IR spectra were recorded as deposit glass film on a Thermo Nicolet 5700 FT-IR spectrometer (Madison, WI, USA). UV spectra were measured in MeCN and MeOH on a Jasco V-530 spectrophotometer (Jasco, Tokyo, Japan). ECD spectra were recorded on a Jasco J-715 (Jasco Co., Tokyo, Japan) spectropolarimeter in MeCN in the range of 200 to 700 nm with the following conditions: scanning speed of 100 nm min^−1^, step size of 0.2 nm, bandwidth of 2 nm, response time of 0.5 s, and accumulation of 16 scans. The spectra were background–corrected using the spectra of MeCN recorded under the same conditions. IR/VCD spectra were recorded on a Jasco FVS–6000 VCD spectrometer (Jasco, Tokyo, Japan) using a resolution of 4 cm^−1^ in the range of 2000 to 900 cm^−1^ in CD_3_OD solutions for 2 h (4000 accumulations). The spectra were background–corrected using spectra of CD_3_OD recorded under the same conditions. ^1^H- and ^13^C-NMR spectra were recorded at 500 and 400, and at 125 and 100 MHz, respectively, in CDCl_3_ on Varian (Varian, Palo Alto, USA) and Bruker (Bruker, Karlsruhe, Germany) spectrometers. The same solvent was used as internal standard. Carbon multiplicities were determined by DEPT (Distortionless Enhancement by Polarization Transfer) spectra [13]. Band-selective ROESY (Rotating-frame nuclear Overhauser Effect correlation Spectroscopy) experiments were performed with the following parameters: mixing time, 200–500 ms; band-selective pulse, 5.74 ms; f1 pulse band-width, 700 Hz. HRESIMS (High Resolution ElectroSpray Ionization Mass Spectroscopy) and ESIMS (ElectroSpray Ionization Mass Spectroscopy), spectra were recorded on an Agilent 6120 Quadrupole LC/MS instrument (Agilent Technologies, Milan, Italy), respectively. Analytical and preparative TLC (Thin Layer Chromatography) were performed on silica gel (Kieselgel 60, F_254_, 0.25 and 0.5 mm respectively) and on reversed phase (Merck, Kieselgel 60 RP-18, F_254_, 0.20 mm) plates. The spots were visualized by exposure to UV radiation (253 nm), or iodine vapour, or by spraying first with 10% H_2_SO_4_ in MeOH and then with 5% phosphomolybdic acid in EtOH, followed by heating at 110 °C for 10 min. Column chromatography was performed using silica gel (Merck, Kieselgel 60, 0.06–0.200 mm).

### 2.2. Fungal Strain

The *R. capillus-albis* strain used in this study was obtained from a diseased *B. tectorum* plant collected by J.F. Pearce at the Whiterocks study site in Skull Valley, Tooele County, Utah, USA (40.329381°–112.778614°, 1447 m) in May 2012.

### 2.3. Extraction and Purification of R. capillus-albis Secondary Metabolites 

The fungus was produced in solid culture by inoculating autoclaved oat chaff [13] with a 5-day-old liquid culture (potato dextrose broth, 22 °C) and incubating under sterile conditions in aluminium pans with plastic covers for three weeks. The culture was then spread out to air-dry in a laminar flow hood for at least several weeks prior to extraction. The dried material (1000 g) was minced using a laboratory mill and extracted with 2 L of MeOH-H_2_O (1% NaCl) (1:1). The mixture was centrifuged for 1 h at 7000 rpm and the supernatant was pooled and defatted by *n*-hexane (2 × 1 L). The resulting aqueous phase was extracted (3 × 1 L) with CH_2_Cl_2_. The combined organic extracts were dehydrated (by Na_2_SO_4_) and evaporated under reduced pressure to yield a brown solid residue (1.42 g). This latter was fractioned by column chromatography on silica gel eluted with CHCl_3_-*i*-PrOH (95:5). Twelve fraction groups were collected on the basis of similar TLC profiles. The residue (26.7 mg) of the third fraction was further purified by TLC eluted with CHCl_3_-*i*-PrOH (99:1) yielding an amorphous solid, identified as reported below as the *trans*-methyl-*p*-coumarate (**4**, 8.3 mg, R*_f_* 0.13). The residue (48.1 mg) of the fifth fraction was purified by TLC successively eluted three times with petroleum ether−Me_2_CO (7:3) affording a yellow amorphous solid and a red powder, identified as reported below as 8-*O*-methylnectriafurone (**3**, 5.9 mg, R*_f_* 0.26) and 9-*O*-methylbostrycoidin (**2**, 12.2 mg, R*_f_* 0.45), respectively. The residue (83.8 mg) of the sixth fraction was further purified by column chromatography on silica gel eluted with CHCl_3_-MeOH (95:5) affording an additional amount of **2** (10.0 mg for a total of 22.2 mg) and the main metabolite as an amorphous red solid, identified as reported below as 9-*O*-methylfusarubin (**1**, 43.7 mg, R*_f_* 0.48). The residue (41.3 mg) of the eighth fraction was further purified by preparative TLC eluted with EtOAc-MeOH-H_2_O (90:7:3), followed by a second purification using petroleum ether-Me_2_CO (7:3) to afford an amorphous solid, identified as reported below as terpestacin (**5**, 7.5 mg, R*_f_* 0.38). 

### 2.4. Compound Characterization

*9-O-Methylfusarubin* (**1**): Its ^1^H- and ^13^C-NMR spectra were very similar to those already reported in literature [14,15]; HRESIMS (+) *m*/*z* 321.0979 [M + H]^+^ (calcd. for C_16_H_17_O_7_, 321.0974).

*9-O-Methylbostrycoidin* (**2**): Its ^1^H- and ^13^C-NMR spectra were very similar to those already reported in literature [14,16]; HRESIMS (+) *m*/*z*: 300.0880 [M + H]^+^ (calcd for C_16_H_14_NO_5_ 300.0872).

*5-O-Methylnectriafurone* (**3**): Its ^1^H- and ^13^C-NMR spectra were very similar to those already reported in literature [14,17]; HRESIMS (+) *m*/*z*: 319.0803 [M + H]^+^ (calcd. for C_16_H_15_O_7_, 319.0818).

*trans-Methyl-p-coumarate* (**4**): Its ^1^H- and ^13^C-NMR spectra were very similar to those already reported in literature [18]; HRESIMS (+) *m*/*z*: 179.0711 [M + H]^+^ (calcd. for C_10_H_11_O_3_, 179.0708).

*Terpestacin* (**5**): ${\left[\alpha \right]}_{D}^{25}$: –17.7 (*c* = 0.4, CHCl_3_); Its ^1^H- and ^13^C-NMR spectra were very similar to those already reported in literature [19]; HRESIMS (+) *m*/*z*: 425.2743 [M + Na]^+^ (calcd. for C_25_H_38_NaO_4_, 425.2750).

*1’-O-(*S*)-α-Methoxy-α-trifluoromethyl-α-phenylacetate (MTPA) ester of5-O-Me nectriafurone* (**6**): (*R*)-(−)-MPTA-Cl (5 μL) was added to **3** (0.5 mg) dissolved in dry pyridine (20 μL). The mixture was kept at room temperature for 24 h and then the reaction was stopped by adding MeOH and the azeotrope formed by addition of C_6_H_6_ was evaporated under reduced pressure. The residue (0.8 mg) was purified by preparative TLC, eluted with CHCl_3_-*i*-PrOH (98:2), yielding **6** as a yellow amorphous solid (*R_f_* 0.43, 0.6 mg). It had: IR ν_max_ 3270, 1732, 1637, 1554, 1453, 1376, 1250 cm^−1^; UV λ_max_ nm (log ε) 444 (4.01), 318 (3.92), 261 (shoulder, 4.16), 237 (4.30); ^1^H-NMR spectrum see Table 1; HRESIMS (+) *m*/*z* 535.1220 [M + H]^+^ (calcd. for C_26_H_22_F_3_O_9_, 535.1216). 

*1’-O-(R)-α-Methoxy-α-trifluoromethyl-α-phenylacetate (MTPA) ester of5-O-Me nectriafurone* (**7**): (*S*)-(+)-MPTA-Cl (5 μL) was added to **3** (0.5 mg) dissolved in dry pyridine (20 μL). The reaction was carried out under the same conditions used for preparing **6** from **3**. The purification of the crude residue (0.7 mg) by preparative TLC eluted with CHCl_3_-*i*-PrOH (98:2), to give **7** as a yellow amorphous solid (*R_f_* 0.43, 0.5 mg). It had: IR ν_max_ 3272, 1733, 1642, 1556, 1462, 1374, 1251 cm^−1^; UV λ_max_ nm (log ε) 443 (4.00), 316 (3.89), 258 (shoulder, 4.14), 238 (4.28); ^1^H-NMR spectrum see Table 1; HRESIMS (+) *m*/*z* 535.1219 [M + H]^+^ (calcd. for C_26_H_22_F_3_O_9_, 535.1216).

### 2.5. Computational Section

Molecular mechanics and preliminary Density Functional Theory (DFT) calculations were run with Spartan’16 (Wavenfunction, Irvine, CA, USA, 2016) with default parameters, default grids and convergence criteria; DFT and Time-Dependent DFT (TDDFT) calculations were run with Gaussian’16 (Gaussian Inc.: Wallingford, CT, USA) [20] with default grids and convergence criteria. Conformational analyses were run with the Monte Carlo procedure implemented in Spartan’16 using Merck molecular force field (MMFF). All structures obtained thereof were optimized with DFT at ωB97X-D/6-31G(d) level in vacuo, and reoptimized at the ωB97X-D/6-311+G(d,p) level in vacuo. Final optimizations were run at the ωB97X-D/6-311+G(d,p) level including the polarizable continuum model (PCM) for MeCN or MeOH in its Integral Equation Formalism (IEF) formulation. The above procedure afforded 3 minima for compound **1**, the most stable of which had a population of >93% in both solvents. Frequency calculations were run at the ωB97X-D/6-311+G(d,p)/PCM level of theory. TDDFT calculations were run with several functionals (B3LYP, CAM-B3LYP, ωB97X-D, M06-2X, PBE0 and BH&HLYP) and def2-TZVP basis set, and included PCM for MeCN. Average ECD spectra were computed by weighting ECD and VCD spectra (calculated for each conformer) with Boltzmann factors at 300K estimated from DFT internal energies. ECD and VCD spectra were plotted using the program SpecDis [21,22], applying the dipole-length formalism for rotational strengths; the difference with dipole-velocity values was negligible. Similarity factors were also estimated with SpecDis.

### 2.6. Bioassays 

The seedling radicle/coleoptile bioassay was conducted using nondormant *B. tectorum* seeds. Compounds were first dissolved in DMSO, which was then diluted to a 2% aqueous solution to obtain each compound at a concentration of 10^−4^ M. For each compound, 250 μL of the solution was pipetted into each of three 3.5 cm Petri dishes onto the surface of one filter paper. Seeds were incubated in 2% DMSO in the control treatment. Four *B. tectorum* seeds were arranged onto the surface of each filter paper in a pattern that made it possible to track individual seeds. Petri dishes were sealed with parafilm to retard moisture loss and incubated at 20 °C with a 12:12 h photoperiod. Germination was scored each day, and germination day was tracked individually for each seed. Five days after germination for each seed, the coleoptile and radicle lengths of each seedling were measured and recorded using electronic calipers. All seeds germinated within 14 days. Petri dish was treated as the blocking variable with individual seeds as replicates within blocks in mixed model analysis of variance (ANOVA) using SAS Proc Mixed (Statistical Analysis Institute, Boca Raton, FL, USA) with compound as the class variable. Response variables (radicle length and coleoptile length) were log-transformed to meet the assumptions of ANOVA prior to analysis.

For the juvenile plant immersion bioassay, plants were grown from seeds to the two-leaf stage in solution culture with a complete nutrient solution. Test compounds were first dissolved in MeOH, and then diluted with H_2_O to 2% MeOH (10^−4^ M concentration of each compound). For each of three replicates of each compound, 2 mL of test solution were pipetted into a small vial. Juvenile plants were then transferred in groups of three to each vial for a total of nine seedlings per treatment. Roots were immersed in the test solution, while shoots were not immersed. The 2% MeOH solvent was used as the control. Vials were placed under fluorescent lights at room temperature (22 °C) and monitored for signs of toxicity over a period from 4–10 days. Ten-day data are reported. The individual plants were scored on a semi-quantitative scale from 0 (no visible damage) to 5 (complete mortality). The ordinal damage scale was approximately linear and was treated as a quantitative response variable in ANOVA using a mixed model procedure for a randomized block design, with vial as the blocking random variable and individual seedlings as replicates within blocks, as described earlier for the radicle/coleoptile elongation test.

## 3. Results and Discussion

**Figure 1:**
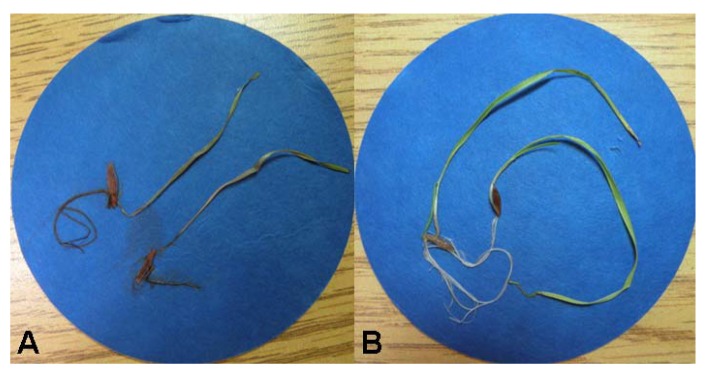
Juvenile plant immersion test on *Bromus tectorum*: (**A**) cheatgrass plants tested with CH_2_Cl_2_ organic extract at 2 mg/mL after 10 days; (**B**) control.

**Figure 2:**
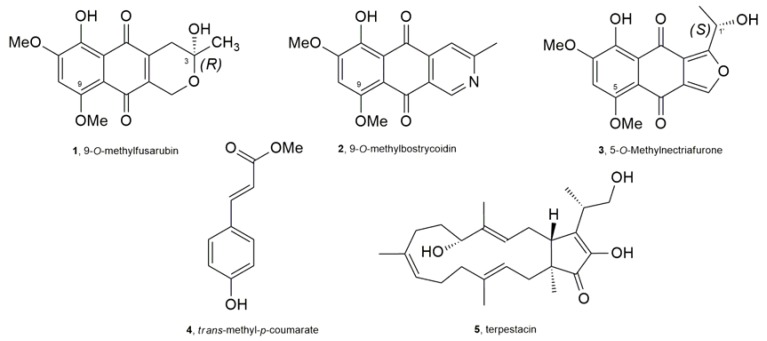
Structures of compounds **1**–**5**.

The solid culture of *R. capillus-albis* was extracted as reported in detail in the Experimental section. The CH_2_Cl_2_ organic extract showed strong phytotoxic activity when tested against *B. tectorum* using an immersion bioassay (Figure 1). Thus, it was purified using a systematic bioassay-guided fractionation (Scheme S1) yielding five pure compounds (**1**–**5**, Figure 2).

Their identification was carried out using extensive spectroscopic, optical and computational methods. In particular, the preliminary ^1^H- and ^13^C-NMR investigation of the main metabolite, obtained as an amorphous red solid, showed the typical signal systems of a polysubstituted naphthoquinone [23,24]. They also appeared to be were very similar to those reported in literature for 9-*O*-methylfusarubin (**1**, Figure 2) [14,15]. This result was also confirmed by HRESIMS, recorded in positive modality, which showed the protonated form [M + H]^+^ at *m*/*z* 321.0979 consistent with a molecular formula of C_16_H_16_O_7_. This metabolite was isolated for the first time from *Fusarium moniliforme* in 1979 [16] and successively from other different *Fusarium* spp. [14,15,25,26], from the endolichenic fungus *Corynespora* sp. BA-10763 [27], and from the sea hare associated fungus *Torula herbarum* [28]. It was also called 8-*O*-methylfusarubin [15], but, in this paper, the name 9-*O*-methylfusarubin is adopted in agreement with IUPAC carbon skeleton numbering. Furthermore, to the best of our knowledge, the absolute configuration (AC) at C-3 of **1** has not been previously determined. Considering that the absolute configuration of natural products is often closely related to their biological activity [10], the AC of **1** and **3** was determined using different methods as following.

**Figure 3:**
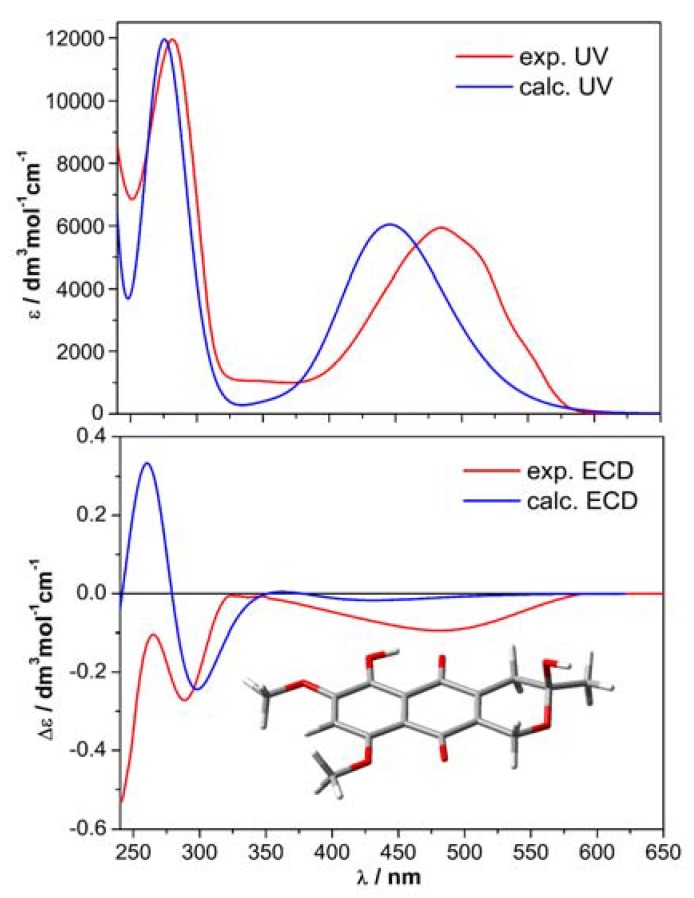
Comparison of experimental and calculated UV/ECD spectra for (*R*)-**1**. Measurement run on 0.25 mM solution in MeCN. Calculations run at CAM-B3LYP/def2-TZVP//ωB97X-D/6-311+G(d,p) level including a PCM solvent model for MeCN; Boltzmann averaged over three conformers (the most stable is shown in the inset); plotted as sum of Gaussian function with 0.5 eV exponential band-width, red-shifted by 21 nm and scaled by a factor 9.835.

The AC of compound **1** was established by quantum-chemical calculations of ECD and VCD spectra, using a consolidated protocol [29,30]. Briefly, the molecular structure was investigated by means of a conformational search with Merck Molecular Force Field (MMFF) and geometry optimizations with density functional theory (DFT) at the ωB97X-D/6-311+G(d,p), including a continuum solvent model (PCM) for MeCN or MeOH. Only three low-energy minima were detected, the most stable of which (see inset in Figure 3) accounted for >93% population at 300K according to internal energies.

**Figure 4:**
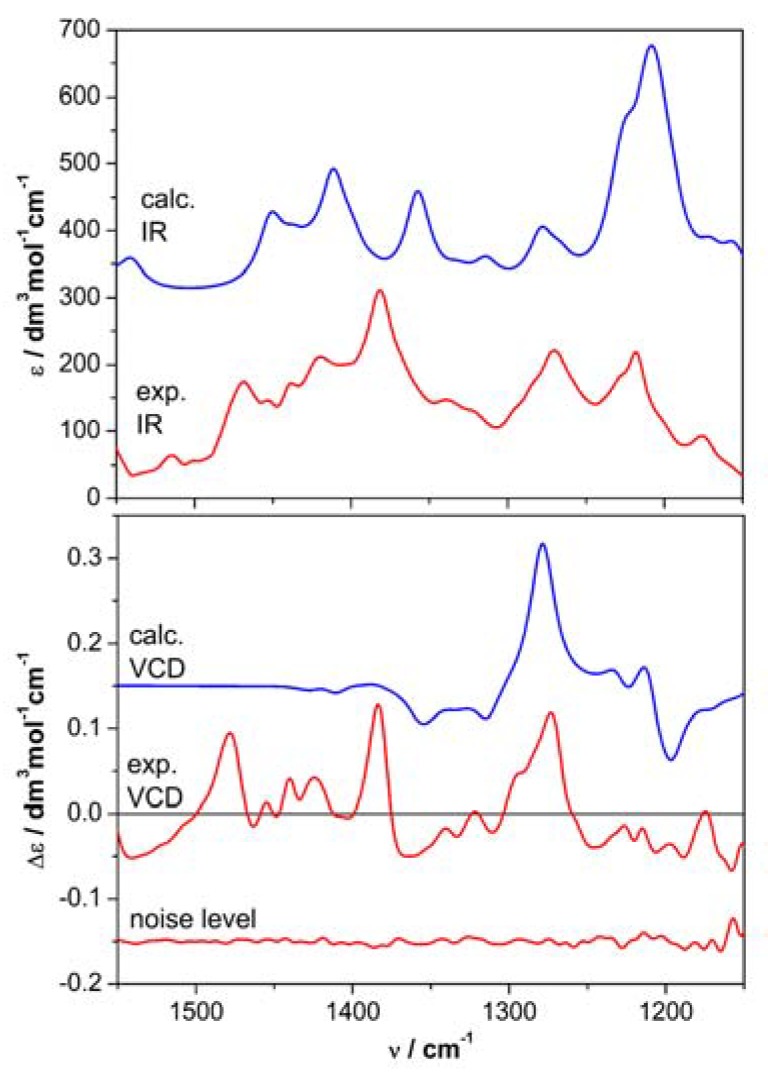
Comparison of experimental and calculated IR/VCD spectra for (*R*)-**1**. Measurement run on 16 mM solution in CD_3_OD, 100 μm BaF_2_ cell. Calculations run at ωB97X-D/6-311+G(d,p) level including PCM solvent model for MeOH; Boltzmann averaged over three conformers; plotted as sum of Lorentzian functions with 10 cm^–1^ half-width at full maximum and frequency scale factor 0.948.

Time-dependent DFT calculations (TDDFT) were run with various combinations of functionals and basis sets; the results obtained with CAM-B3LYP/def2-TZVP/PCM are discussed here. Additionally, frequency calculations were run at the ωB97X-D/6-311+G(d,p)/PCM level. Figure 3 and Figure 4 show, respectively, the experimental and calculated UV/ECD and IR/VCD spectra for the (3*R*) enantiomer of **1**. Neither ECD nor VCD spectra were perfectly reproduced over the whole available range; however, the agreement was in both cases sufficient and consistent. The similarity factors [21,22] were estimated as 0.57 for (*R*) enantiomer and 0.037 for the (*S*) enantiomers by VCD. The use of two chiroptical techniques, whose conclusions reinforce each other, is especially important when dealing with weak ECD or VCD spectra, as in the current and similar cases [30,31]. Thus, the absolute configuration of 9-*O*-methylfusarubin is assigned as (*R*)-**1**. The consistency between experimental and calculated *g*-factors (Δε/ε) in the VCD/IR spectra indicates the enantiomeric purity of the natural product **1**.

9-*O*-methylfusarubin (**1**) showed bactericidal activity against some Gram+ bacteria while it was inactive when tested against Gram- bacteria [25,32]. Other biological activities, namely phytotoxic and chlorotic-inducing properties in plants as well as its potential as an anticancer agent, were evaluated when it was isolated from *Fusarium oxysporum*. It showed phytotoxic activity against wheat, bean, corn and tobacco plants at different concentrations and antineoplastic effects on *ras* transformed liver epithelial cells [26]. Finally, compound **1** inhibited hepatic glucose production at an IC_50_ value of 4.8 μM and showed cytotoxicity against hepatic cells after incubation for 48 h [33].

Compound **2** was identified as the known metabolite 9-*O*-methylbostrycoidin by comparing its ^1^H- and ^13^C-NMR spectra with those reported in literature when it was isolated from some species of *Fusarium* [14,16]. Its HRESIMS, recorded in positive modality, showed the protonated form [M + H]^+^ at *m*/*z* 300.0880 consistent with a molecular formula of C_16_H_13_NO_5_. 9-*O*-methylbostrycoidin was also isolated from the liquid cultures of the mangrove endophytic fungus *Aspergillus terreus* (No. GX7-3B) [34] and of the endolichenic fungus *Corynespora* sp. BA-10763 [27]. In addition, **2** displayed remarkable inhibiting action against α-acetylcholinesterase (AChE) with an IC_50_ value of 6.71 μM [34], inhibited hepatic glucose production at an IC_50_ value of 30 μM, and showed weak cytotoxicity against hepatic cells after incubation for 48h [33]. It also showed bactericidal activity against some Gram+ bacteria, but it has never been tested for phytotoxic activity.

**Figure 5:**
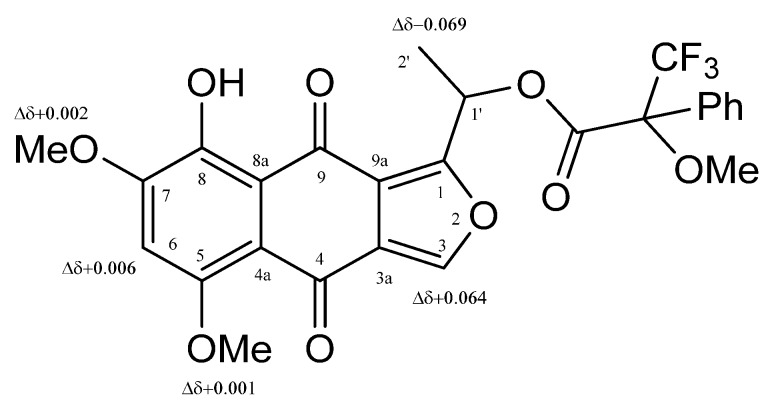
Structures of 1’-*O*-*S*- and 1’-*O*-*R*-MTPA of **3** (**6** and **7**, respectively), reporting the Δδ values obtained by comparison of each proton system.

Compound **3** showed ^1^H- and ^13^C-NMR data very similar to those previously reported in literature for the 5-*O*-methylnectriafurone [14,17]. This identification was supported by the data of its HRESIMS, which showed the protonated form [M + H]^+^ at *m*/*z* 319.0803 consistent with a molecular formula of C_16_H_14_O_7_. This compound was only produced by *F. oxysporum* obtained from roots of diseased citrus trees [17] and by a strain of *Fusarium proliferatum*, an endophytic fungus isolated from *Syzygium cordatum* [14]. No biological activities were reported and its AC was not determined. Thus, the AC of **3** was determined applying the advanced Mosher’s method [19] appropriately derivatizing the chiral secondary alcohol at C-1’. In particular, 5-*O*-methylnectriafurone (**3**) by reaction with *R*-(−)-α-methoxy-α-trifluoromethylphenylacetyl (MTPA) and *S*-(+)-MTPA chlorides, was converted into the corresponding diastereomeric esters at C-1’ (**6**, **7**). Subtracting the proton chemical shifts (Table 1) of the 1’-*O*-*R*-MTPA (**7**) from those of 1’-*O*-*S*-MTPA (**6**) esters, the Δδ (**6**–**7**) values for all of the protons were determined as reported in Figure 5. The positive Δδ values were located on the right side, and those with negative values on the left side of model A as reported in Cimmino et al. [35]. This model allowed the assignment of the *S* configuration at C-1’. The appearance of single set of signals for both **6** and **7** indicates the enantiomeric purity of the natural product **3**. 

On the basis of these findings, 5-*O*-methylnectriafurone was formulated as (1’*S*)-1-(1-hydroxyethyl)-5,7,8-trimethoxynaphtho[2,3-*c*]furan-4,9-dione (**3**). 

Compound **4** was identified as the methyl ester of *p*-coumaric acid by comparing its ^1^H- and ^13^C-NMR data with those reported in the literature [18] and confirmed by its HRESIMS which showed the protonated form [M + H]^+^ at *m*/*z* 179.0711 consistent with a molecular formula of C_10_H_10_O_3_. Compound **4** is a well-known plant metabolite with diverse biological activity that has rarely been reported as a fungal metabolite [36]. However, phytotoxic activity was reported only against the green alga *Selenastrum capricornutum* when compound **4** was isolated from the aquatic plant *Schoenoplectus lacustris* [37].

Compound **5** showed ^1^H- and ^13^C-NMR data very similar to those reported in the literature for terpestacin [19]. Furthermore, the measured optical rotation value is coincident with the previously reported one, indicating the enantiomeric purity of the natural product **5** [19]. Its identification was supported by the data of the HRESIMS, which showed the sodium cluster [M + Na]^+^ at *m*/*z* 425.2743 consistent with a molecular formula of C_25_H_38_O_4_. Terpestacin (**5**) is a well-known mycotoxin that was first isolated in 1993 from *Arthrinium* sp. FA 1744 (ATCC 74132) and showed syncytium formation inhibitory activity [38]. Moreover **5**, together with its analogue fusaproliferin, was recently isolated from a mangrove-derived endophytic fungus *F. proliferatum* MA-84 [39] and from the fimicolous fungi *Cleistothelebolus nipigonensis* and *Neogymnomyces virgineus* [19]. Furthermore, some key hemisynthetic derivatives were prepared starting form terpestacin and fusaproliferin and their antifungal activity was tested against some Ascomycetous fungi. In particular, these metabolites and their derivatives reduced the growth of *Alternaria brassicicola*, *Botrytis cinerea* and *Fusarium graminearum*, demonstrating their allelopathic activity [19].

**Figure 6:**
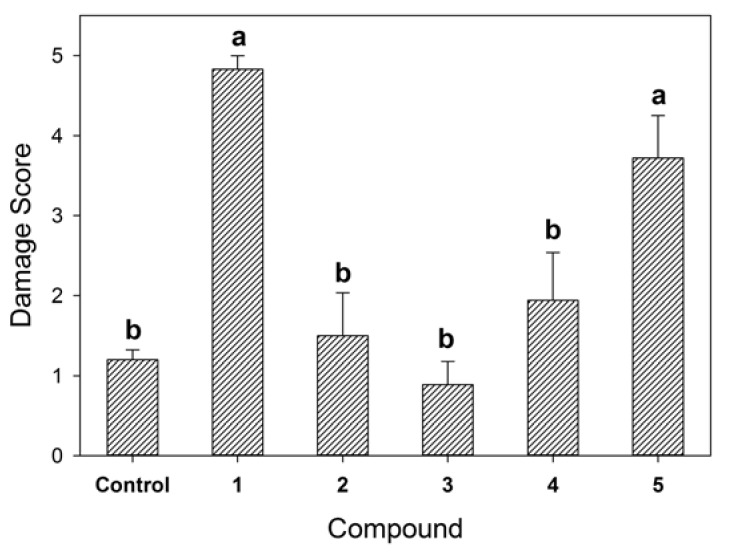
Effect of five pure compounds obtained from *R. capillus-albis* solid culture on juvenile plants of *B. tectorum* when tested at 10^−4^ M in a juvenile plant immersion bioassay against a methanol solvent control. Symptoms were scored at 10 days on an approximately linear scale from 0 (no visible damage) to 5 (complete mortality). Bars headed by the same letter (a or b) are not significantly different at *p* < 0.05 according to a means separation test from analysis of variance.

The five pure compounds were tested for toxicity against *B. tectorum* using a radicle/coleoptile elongation test and a juvenile plant immersion test at 10^−4^ M as described in the Experimental section. None of the compounds slowed or prevented seed germination or had a negative effect on 5-day post-germination length of either the radicle or the coleoptile relative to the control treatment (data not shown). In the juvenile plant immersion test (Figure 6), 9-*O*-methyl-fusarubin and terpestacin showed significant phytotoxic effects relative to the control. With these two compounds, most juvenile plants suffered complete wilting, discoloration, and death within 10 days as illustrated in Figure 1. Both metabolites have potential as natural herbicides. However, before planning their practical application, it is important to investigate their persistence in soil and water and toxicity of their degraded compounds.

## 4. Conclusions

This paper reports the isolation and identification 9-*O*-methylfusarubin, 9-*O*-methylbostrycoidin, 5-*O*-methylnectriafurone, *trans*-methyl-*p*-coumarate and terpestacin produced as secondary metabolites by the newly described plant pathogen *R. capillus-albis*, as well as their phytotoxic activity against *B. tectorum*. The absolute configuration at C-3 of 9-*O*-methylfusarubin and at C-1’ of 5-*O*-methylnectriafurone were also determined for the first time by applying electronic circular dichroism (ECD), vibrational CD (VCD), computational methods, and the advanced Mosher’s method, respectively. The results obtained with 9-*O*-methylfusarubin and terpestacin in a juvenile plant immersion bioassay suggest that these two compounds could play a role in pathogenesis on *B. tectorum*. However, there may be additional roles for these compounds beyond those investigated in this study. Compounds **1**–**3** could be responsible for the coloration of the fungal stromata as already demonstrated for the fruiting bodies in *Fusarium fujikuroi* [40]. In fact, they have not been detected in liquid PDB culture of *R. capillus-albis* (Masi et al. unpublished data), supporting this idea. Furthermore, these naphthoquinones could be protectants against harmful environmental conditions such as UV irradiation, reactive oxygen species (ROS) and desiccation [40,41] or help the fungus in defense against its natural enemies. The results of the bioassays and these last considerations could increase the potential of *R. capillus-albis* to control cheatgrass. Finally, considering several biological properties showed by natural anthraquinones [42], future studies will be performed to evaluate the potential practical application of these compounds in different fields. In particular, it will be interesting to speculate on their industrial applications as natural dyes to replace synthetic chemicals in formulation in order to prevent negative environmental effects [42].

## Figures and Tables

**Table 1:** ^1^H-NMR data of **6** and **7** recoded in CDCl_3_^a.^

	6	7
Position	δ_H_ (*J* in Hz)	δ_H_ (*J* in Hz)
3	8.062 (1H, s)	7.998 (1H, s)
MeO-5 ^b^	4.042 (3H, s)	4.041 (3H, s)
6	6.833 (1H, s)	6.827 (1H, s)
MeO-7 ^b^	4.014 (3H, s)	4.012 (3H, s)
1’	5.976 (1H, q, *J* = 8.0)	5.824 (1H, q, *J* = 8.0)
2’	1.717 (3H, d, *J* = 8.0)	1.786 (3H, d, *J* = 8.0)
MeO	3.580 (3H, s)	3.569 (3H, s)
Ph	7.505–7.380 (5H, m)	7.491–7.367 (5H, m)
OH-8	13.481 (br s)	13.480 (br s)

^a^ The chemical shifts are in δ values (ppm) from TMS. ^b^ These signals could be exchangeable.

## Supplementary Materials

A fractionation scheme (Scheme S1) of the fungal metabolites **1**–**5** and their ^1^H-NMR spectra are reported in the Supplementary Materials available online.
