# Supplementary material for: Phytotoxic Activity of Metabolites Isolated from Rutstroemia sp.n., the Causal Agent of Bleach Blonde Syndrome on Cheatgrass (Bromus tectorum)
Source: Molecules. 2018 Jul 16;23(7):1734. doi: 10.3390/molecules23071734 (PMC6100615; doi:10.3390/molecules23071734)

SUPPLEMENTARY MATERIAL

Article

Phytotoxic Activity of Metabolites Isolated from *Rutstroemia sp.n.*, the Causal Agent of Bleach Blonde Syndrome on Cheatgrass (*Bromus Tectorum*)

Marco Masi [1], Susan Meyer [2], Marcin Górecki [3,4], Gennaro Pescitelli [3], Suzette Clement [2], Alessio Cimmino [1] and Antonio Evidente [1,*]

[1] Dipartimento di Scienze Chimiche, Università di Napoli "Federico II", Complesso Universitario Monte S. Angelo, Via Cintia 4, 80126 Napoli, Italy; marco.masi@unina.it (M.M.); alessio.cimmino@unina.it (A.C.)
[2] US Forest Service Rocky Mountain Research Station, Shrub Sciences Laboratory, 735 North 500 East, Provo, UT 84606, USA; semeyer@xmission.com (S.M.); sclement@fs.fed.us (S.C.)
[3] Dipartimento di Chimica e Chimica Industriale, Università di Pisa, Via Moruzzi 13, 56124 Pisa, Italy; gennaro.pescitelli@unipi.it (G.P.); marcin.gorecki@icho.edu.pl (M.G.)
[4] Institute of Organic Chemistry, Polish Academy of Sciences, ul. Kasprzaka 44/52, 01-224 Warsaw, Poland
* Correspondence: evidente@unina.it; Tel.: +39-081-253-9178

Received: 25 June 2018; Accepted: 12 July 2018; Published: date

List of content

Page 2: **Scheme S1**. Fractionation scheme of the fungal metabolites.

Page 3: **Spectra 1**. ^{1}H NMR spectrum of 9-*O*-methylfusarubin (**1**) ($CDCl_3$, 500 MHz).

Page 4: **Spectra 2**. ^{1}H NMR spectrum of 9-*O*-methylbostrycoidin (**2**) $CDCl_3$, 500 MHz).

Page 5: **Spectra 3**. ^{1}H NMR spectrum of 5-*O*-methylnectriafurone (**3**) ($CDCl_3$, 400 MHz).

Page 6: **Spectra 4**. ^{1}H NMR spectrum of *trans*-methyl-*p*-coumarate (**4**) ($CDCl_3$, 500 MHz).

Page 7: **Spectra 5**. ^{1}H NMR spectrum of terpestacin (**5**) ($CDCl_3$, 400 MHz).

Scheme S1. Fractionation scheme of the fungal metabolites.

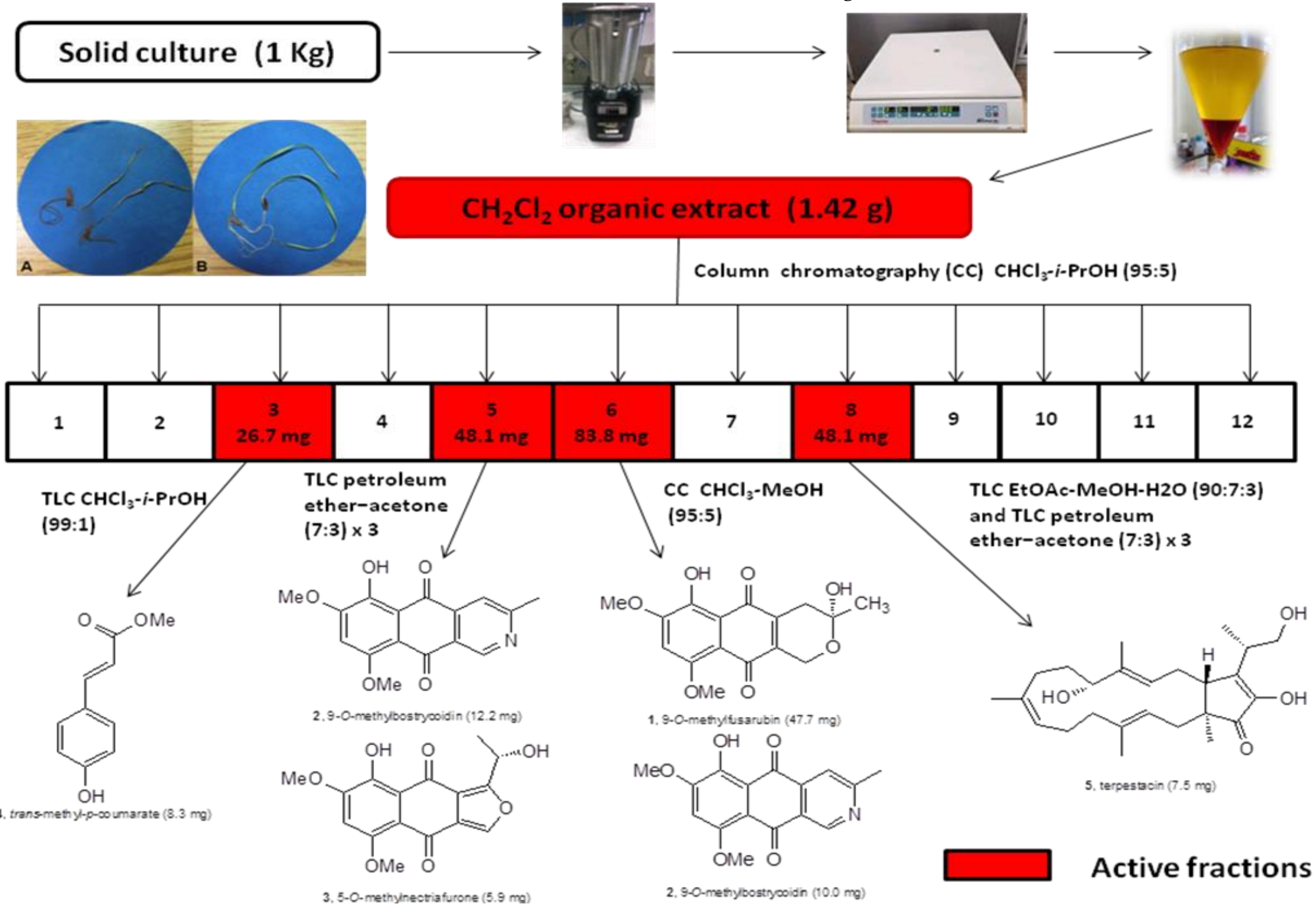

Spectra 1. ^{1}H NMR spectrum of 9-*O*-methylfusarubin (**1**) ($CDCl_3$, 500 MHz).

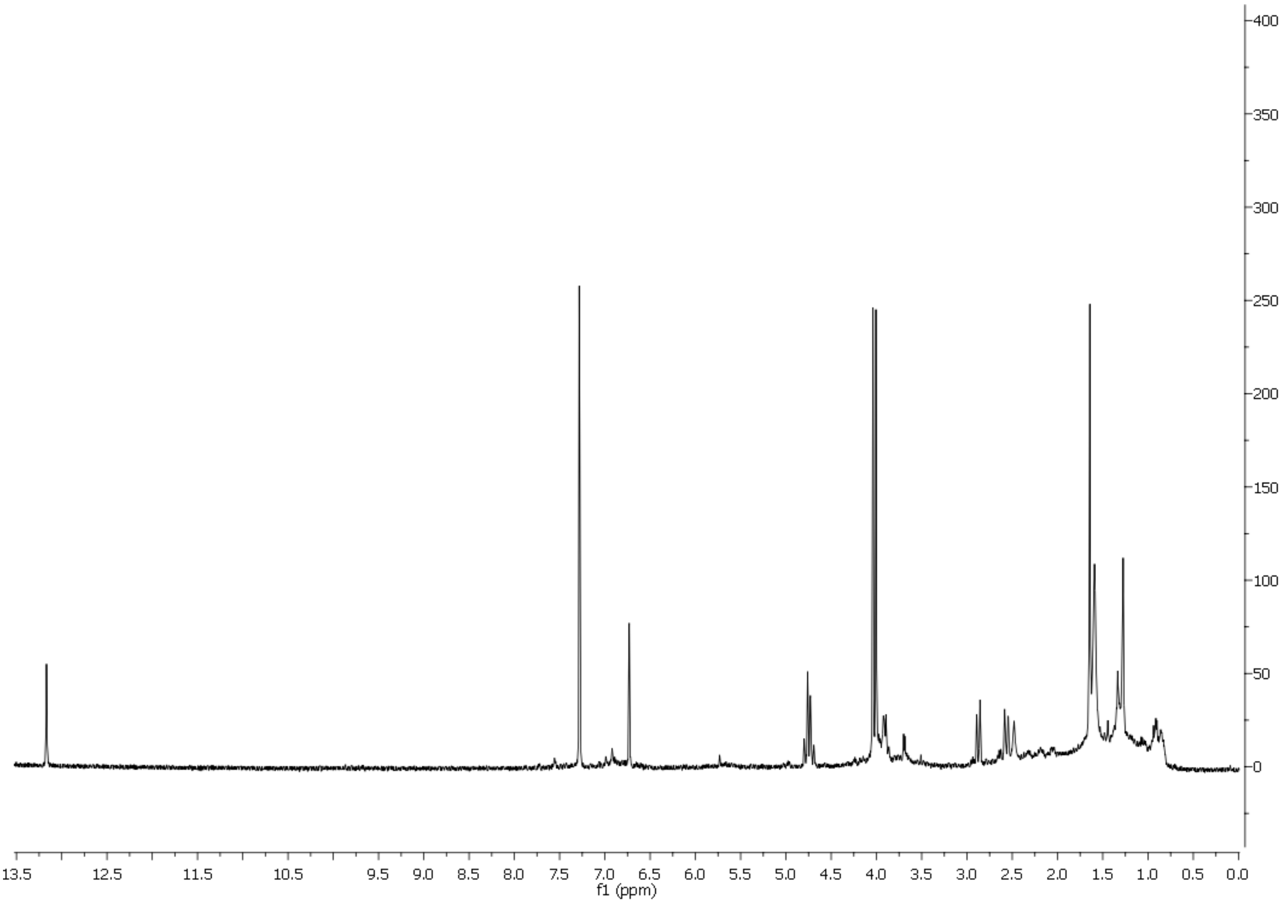

Spectra 2. ^{1}H NMR spectrum of 9-*O*-methylbostrycoidin (**2**) $CDCl_3$, 500 MHz). .

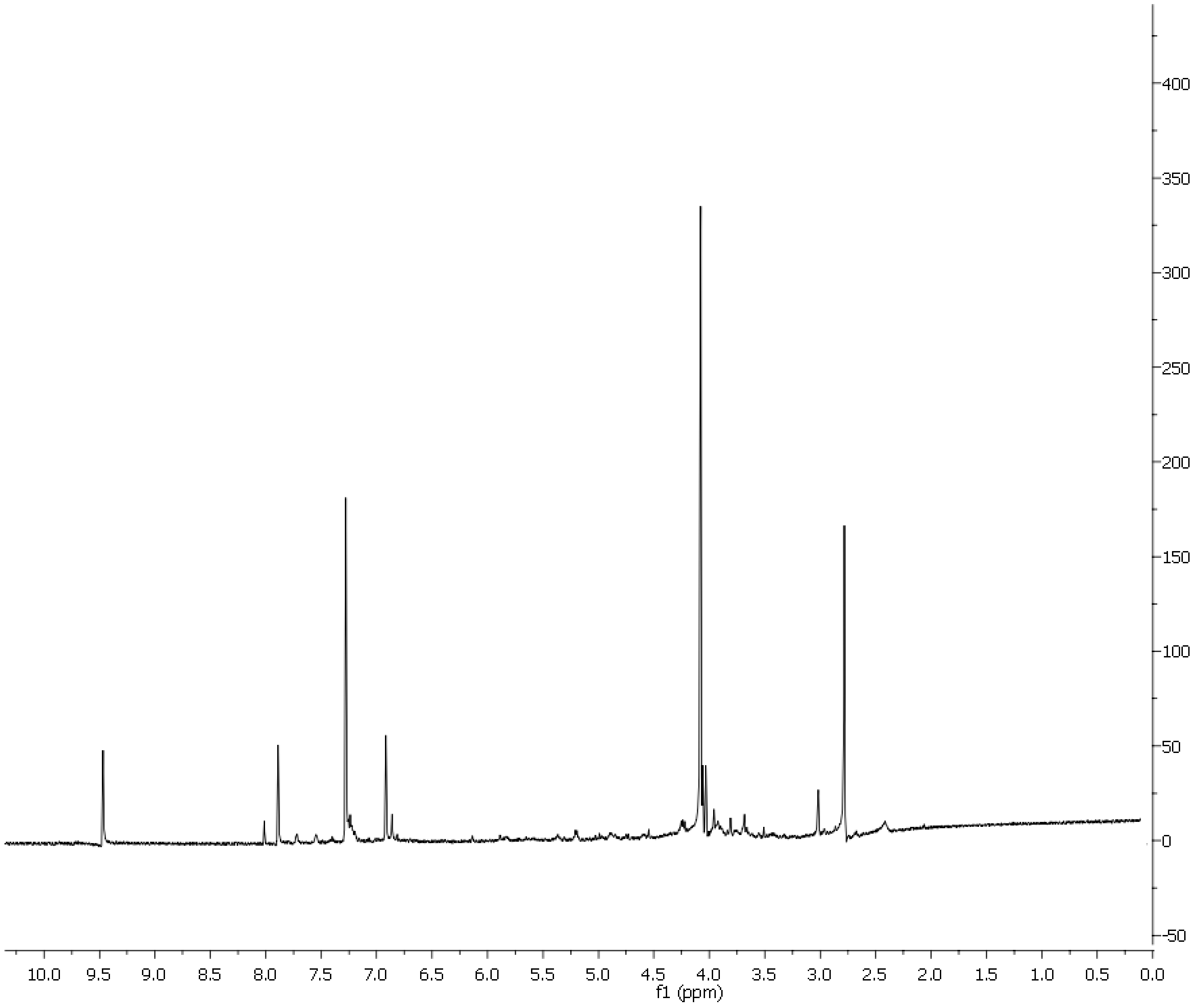

Spectra 3. ^{1}H NMR spectrum of 5-*O*-methylnectriafurone (**3**) ($CDCl_3$, 400 MHz).

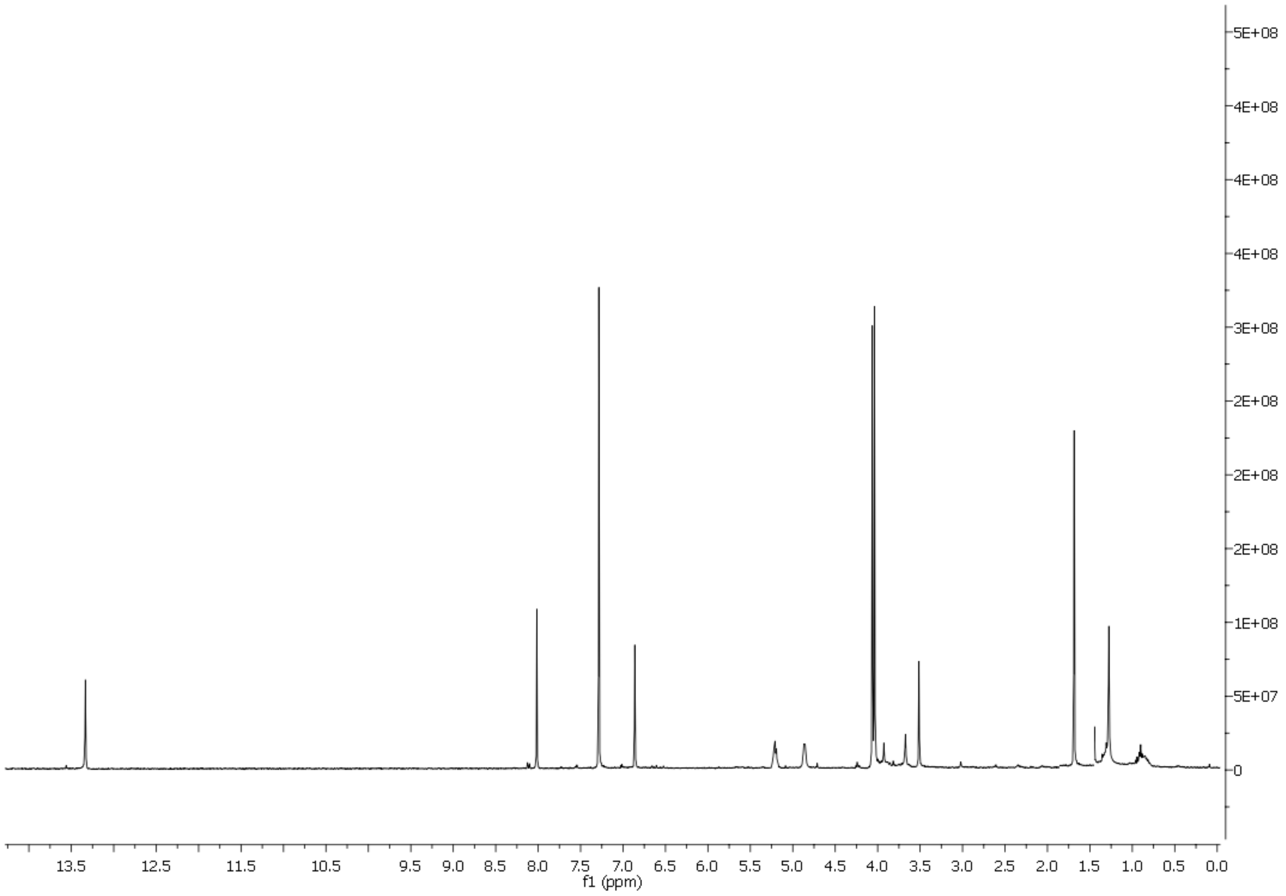

Spectra 4. ^{1}H NMR spectrum of *trans*-methyl-*p*-coumarate (**4**) ($CDCl_3$, 500 MHz).

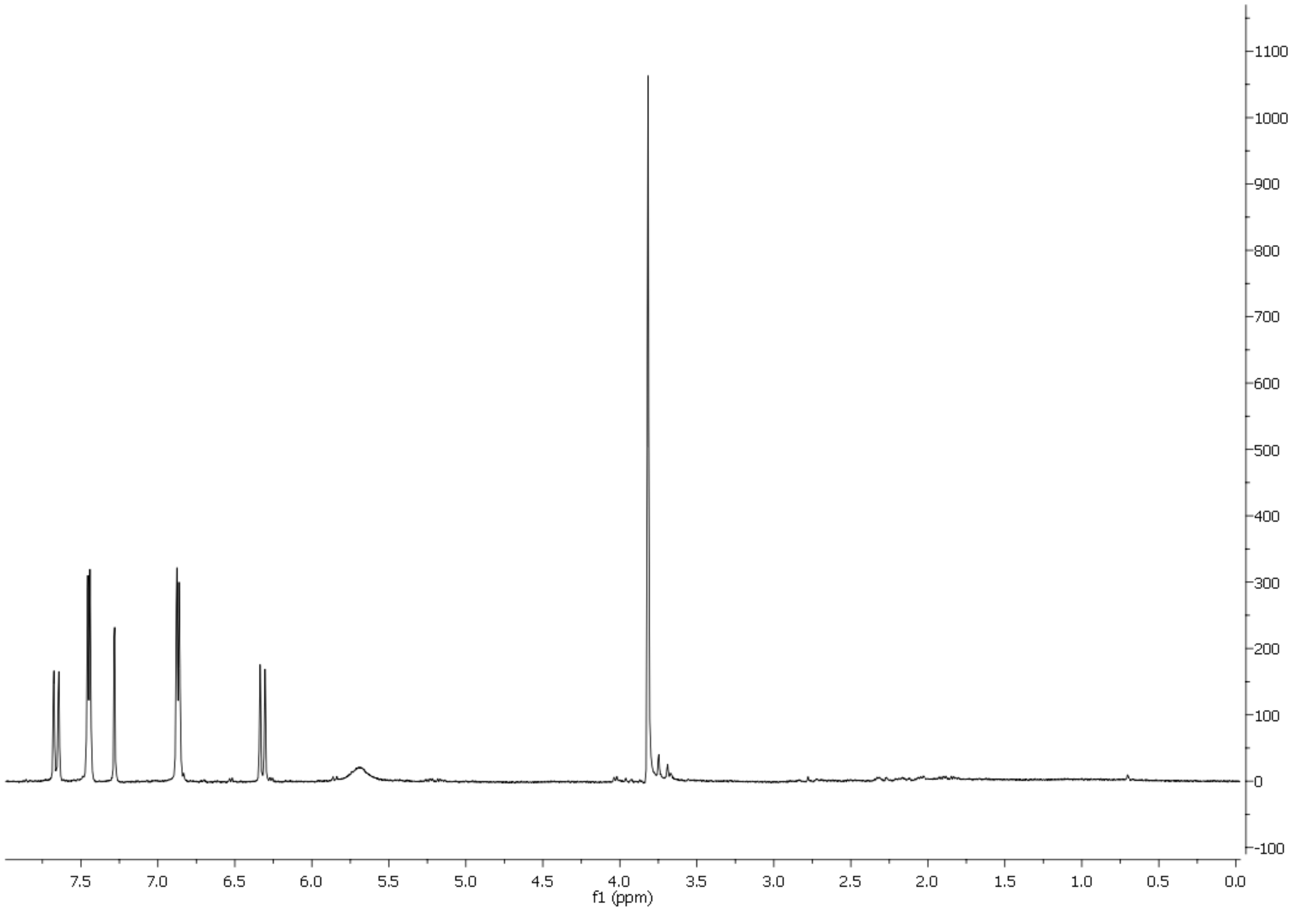

Spectra 5. ^{1}H NMR spectrum of terpestacin (**5**) ($CDCl_3$, 400 MHz).

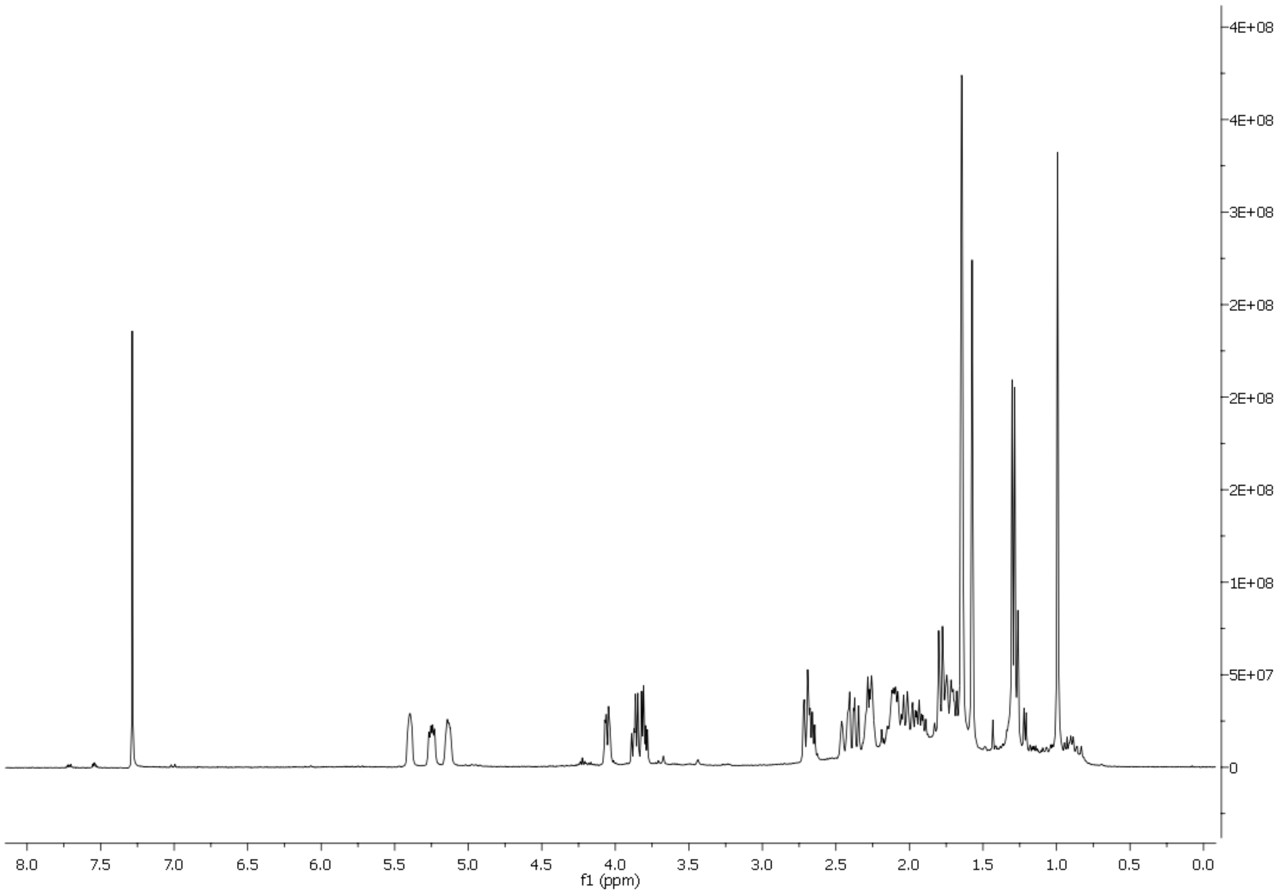

Supplement: Supplementary file 1 [file molecules-23-01734-s001.pdf]
